# European Registry of Hereditary Pancreatic Diseases (EUROPAC): protocol for primary and secondary screening in individuals with inherited pancreatic disease syndromes for pancreatic ductal adenocarcinoma and complications of other pancreatic diseases

**DOI:** 10.1136/bmjopen-2025-100027

**Published:** 2025-04-03

**Authors:** Annabelle Boughey, Phil Hopley, Ioannis Sarantitis, Paul Thomas, Beata Gubacsi, Kayleigh Jevons, Emma Crowe, Eleri Hughes, Isobel Quinn, Liam Royle, Richard J Jackson, William Greenhalf, Chris Halloran

**Affiliations:** 1 Molecular and Clinical Cancer Medicine, University of Liverpool, Liverpool, England, UK; 2 Royal Liverpool University Hospital, Liverpool, UK; 3 Liverpool Clinical Trials Centre, University of Liverpool, Liverpool, UK

**Keywords:** Pancreatic surgery, Pancreatic disease, Cancer genetics, REGISTRIES

## Abstract

**Abstract:**

**Introduction:**

Pancreatic cancer is a devastating disease and one of the top causes of cancer death worldwide. Over 30% of cases are potentially avoidable, and while screening for this disease should be possible, the current methods, without risk stratification to detect high-risk groups, are unlikely to detect these individuals. A tailored screening pathway could be applied to individuals with a germline genetic cause of pancreatic cancer, which may account for around 10% of cases.

**Methods and analysis:**

EUROPAC, although having international reach, is described here in relation to the UK only. This national prospective observational study has run for several decades but was modified into the current trial in 2019, which aims to recruit and screen 10 000 individuals with either familial pancreatic cancer or hereditary pancreatitis (HP). Applicants are assessed for eligibility by generating an individual pedigree and by attributing a family risk score (FR). Individual risk is assessed according to age. Individuals over 40 with an FR >30 are offered baseline imaging and then three yearly triplets of annual endoscopic ultrasound (EUS) and an MRI (in the third year). Those with an FR >60 are offered both EUS and MRI yearly. HP patients are screened by CT and/or MRI dependent on risk stratification using the presence of diabetes, smoking or alcohol consumption. Low-risk (absence of these factors) patients have a CT every 2 years, and high-risk (one or more of the above factors) patients have alternate yearly screening with CT, then MRI. Biospecimens are collected at pragmatic intervals with first sampling at registration to support future biomarker development to detect pancreatic cancer early. Detection of early-stage pancreatic cancer and actionable lesions will be evaluated.

**Ethics and dissemination:**

The EUROPAC study has been reviewed and approved by the Yorkshire and Humber Research Ethics Committee (Ref 19/YH/0250). Study results will be disseminated through national and international symposium presentations and published in peer-reviewed, open-access journals. All participants provided informed consent prior to entering the study.

**Trial registration number:**

ISRCTN62546421

**STRENGTHS AND LIMITATIONS OF THE STUDY**

- Although there is a recommendation for screening familial pancreatic cancer and hereditary pancreatitis families, current guidelines do not specify by whom or where screening should be carried out, the interval of screening, the age at which to start screening or whether risk stratification should be applied to these cohorts.
- Applicants to EUROPAC undergo intensive fact-checking and the development of a family pedigree, allowing risk-stratified screening and decision making.
- Participants will receive screening in regional EUROPAC centres.
- EUROPAC has the potential to reverse the current trend of <20% diagnosed disease at stage I and II to be >70%, in screen-detected cancers, and thus be in line with the national NHS (National Health Service) cancer plan.
- It is estimated that 15% of the 10 000 will have an actionable lesion.

## Introduction

 Pancreatic cancer is a devastating disease and one of the top causes of cancer death worldwide. The 5-year survival rate in the UK is less than 9% with only marginal improvement over the last four decades.^1^,^3^ 75–80% of cases present at stage III and IV, with relatively few cases among the stage III group being brought to surgery by neoadjuvant strategies. The remaining cases are amenable to curative treatment by surgery and adjuvant therapy.^4^ We are a long way off from the National Health Service plan of 75% of tumours presenting at stage I or II. Acute pancreatitis is somewhat less common but still has an incidence of over 10 new cases being diagnosed in 100 000 individuals every year. With a relatively low incident rate, population-wide screening as a route to earlier detection is not justified for Pancreatic Ductal Adenocarcinoma (PDAC).^5^

Familial pancreatic cancer (FPC) is defined as a family with at least two affected first-degree relatives, and an estimated 4–10% of pancreatic cancers diagnosed have a familial background.^6^ FPC appears to be inherited in an autosomal dominant manner, and around 10–13% of families carry germline mutations in BRCA2, PALB2, ATM, CHEK2, CDKN2A, Lynch syndrome mismatch repair genes, Fanconi anaemia related genes and PRSS1 (hereditary pancreatitis (HP)) as well as other novel genes.^7^ Methods to identify FPC and HP groups are needed to aid the development of new screening strategies. The EUROPAC study was set up in 1996 with the principal objective to allow early detection of pancreatic cancer and to improve treatment for pancreatic diseases by prediction of disease progression. The registry currently includes over 50 000 individuals made up of over 2500 families with FPC or HP. Asymptomatic high-risk individuals are offered a secondary screening programme for the early detection of pancreatic cancer. This consists of a baseline CT, then depending on the FR^8^ score either: >30; annual (endoscopic ultrasound (EUS)), blood biomarkers Ca19.9 and HbA1c and MRI once in three years. If FR >60; annual EUS and MRI are provided. Participants with normal screening re-enter the next triplet, while those with abnormalities which require further investigation and/or intervention (actionable lesions) are discussed at regular national EUROPAC multidisciplinary team meetings.

### Methods and analysis

### Study setting

The EUROPAC study is an international observational registry and a secondary screening study, recruiting families with a history of pancreatic cancer or pancreatitis to facilitate the development of a screening pathway for PDAC.

### Dates of the study

The study was started on 01 July 2019. The proposed end of the study is 03 January 2039.

### Study design

Registry (observational) and secondary screening study. The study is comprised of four elements: two registries and two pilot secondary screening programmes.

- The FPC registry.
- The HP registry.
- FPC secondary screening.
- Secondary screening of individuals with pancreatitis and cystic lesions.

**Figure 1:**
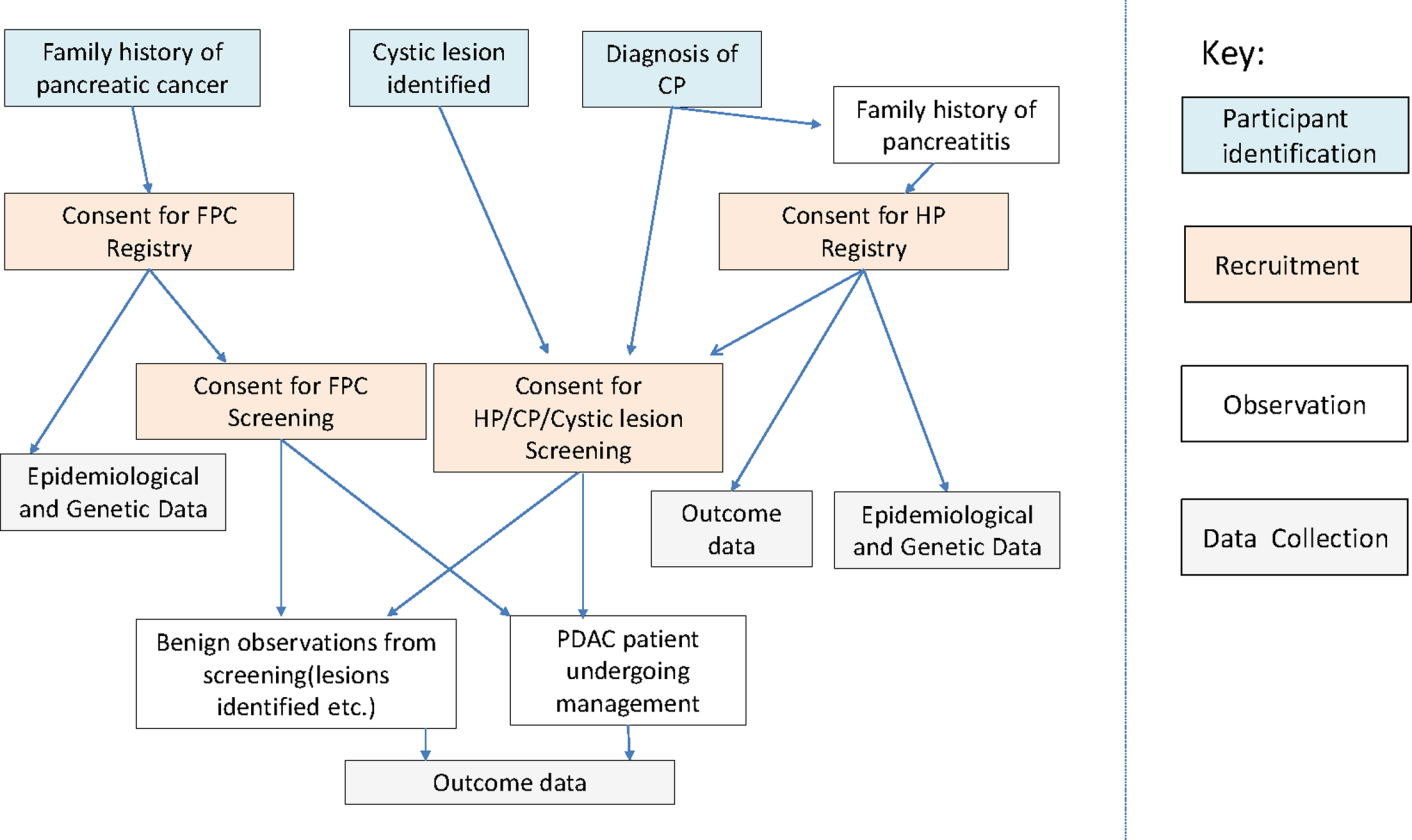
Schema for the European Registry of Hereditary Pancreatic Diseases study showing how participants are recruited and form a part of the study. CP, Chronic Pancreatitis; FCP, familial pancreatic cancer; HP, hereditary pancreatitis.

These four projects generate epidemiological and outcome data. Participants are identified because of a family history of pancreatic cancer or because of a pancreatic condition (pancreatitis or cystic lesions identified by imaging). They are then entered into a registry of high-risk individuals (if appropriate). Individuals are recruited for the secondary screening programme from registries or directly because of pancreatic conditions with worrisome features (figure 1).

## Eligibility criteria

### Inclusion criteria for registry

- Two first-degree relatives, on the same side of the family, with pancreatic adenocarcinoma.
- A family with three or more relatives, on the same side of the family, with pancreatic ductal adenocarcinoma.
- Families with pancreatic cancer and other cancers (eg, bowel, breast/ovarian, melanoma, gastric) that suggest a known cancer predisposition syndrome.
- Families with a known inherited cancer syndrome (eg, hereditary non-polyposis colorectal cancer, familial atypical multiple mole melanoma, Lynch syndrome) with one individual affected by pancreatic cancer.
- Peutz-Jeghers syndrome.
- Families with a causative gene linked to pancreatic cancer (eg, BRCA2 or yet undiscovered genes) and at least one case of pancreatic cancer in the family.
- Families with two or more relatives with idiopathic pancreatitis.
- Families with at least one case of pancreatitis and a confirmed causative mutation in the PRSS1 gene.

### Inclusion criteria for screening

- Individuals over 40 years of age from an established pancreatic cancer family. Inheritance of predisposition consistent with high penetrant autosomal dominant inheritance. For example, at least two first-degree relatives with pancreatic ductal adenocarcinoma, where no non-penetrant carriers have to be assumed over the age of 75.
- Unaffected member of a family with an associated cancer syndrome and at least one case of pancreatic cancer, who has been shown to carry the relevant genetic alteration.
- Any member of a HP family who has been confirmed to carry a causative PRSS1 mutation.
- An affected member of a family consistent with HP who has tested negative for known causative PRSS1 mutations.
- Individuals incidentally found to have cystic lesions or other clinical features that indicate an increased risk of pancreatic cancer may also be included.

### Exclusion criteria

- Any participant who is incapable of providing informed consent.
- For genetic testing: any individual who does not consent to be informed of clinically significant results. Genetic testing for a predisposition for pancreatitis will still be carried out on individuals who have expressed a wish not to be informed following detailed discussions on the limitations of a right not to know in this case; testing will be carried out only if individuals wish to have testing just for research.
- For screening: individuals of less than 40 years of age or 10 years younger than the youngest case in the family will be excluded.
- For screening: any individual deemed to have less than 2% chance of developing PDAC in the next 3 years will be excluded. This will depend on the evidence supporting the models, and the exclusion will only apply if the steering committee agrees on the risk assessment. Risk assessment will be made using progressively developed models.
- For screening: any female participant able to bear a child but who has not taken appropriate contraceptive measures.

### Methods of participant identification

### Study timeline

Eligible individuals will be sent a participant information sheet explaining the EUROPAC registry and a family history questionnaire and consent form (onlinesupplemental appendix files 1(HP)  2(FPC)). They will have the opportunity to ask questions before filling out the documents. The documents will collect a full medical history, drug and surgical history and detailed family history. Blood and urine samples will be taken for plasma and serum research for biobanking according to the Good Clinical Practice (GCP) Laboratory standards to allow for translational research. Those who are identified to be at a higher risk of developing PDAC are offered a secondary screening programme consisting of CT, EUS, MRI and bloods (onlinesupplemental appendix files 3(HP)  4(FPC)). They are sent a secondary screening information sheet and have a clinic appointment with the study research fellow before consenting to the screening programme. Participants will be screened on either a 6-month or 1-year basis, depending on their risk. All participants are followed up on an annual basis. These are summarised in table 1.

**Table 1:** Surveillance intervals: surveillance for hereditary pancreatitis individuals and surveillance for familial pancreatic cancer individuals

Surveillance intensity for HP		CTFrequency	eMRIFrequency
Standard	No Diabetes	Every 2 years	–
	Non-Smoking		
	No Ethanol		
Enhanced	Diabetes or	Alternate 2 years	
	Smoker or		
	Ethanol		

Surveillance Intensity for FPC		EUSFrequency	eMRIFrequency
Standard	Germline mutation	Every 1 year	Every 3 years
	or		
	Risk score>30		
Enhanced	Germline mutation	eMRI and EUS every year	
	and/or		
	Risk score >60		

A baseline CT scan is performed in the first year to check the individual does not already have pancreatic cancer and to be used as reference during surveillance.

Top table is for Hereditary pancreatitis Screening

Bottom Table is for Familial Pancreatic Cancer

CTDual phase pancreas computer tomographyeMRIenhanced magnetic resonance imagingEUSendoscopic ultrasoundFPCFamilial Pancreatic Cancer HPHereditary Pancreatitis

## Objectives

### Primary

The primary objective is to recruit risk-stratified individuals with predisposition to pancreatic cancer into secondary screening, with standardised collection of biomaterials and clinicopathological data, in order to detect premalignant lesions or early-stage pancreatic cancer.

### Secondary

Detection of late-stage pancreatic cancer and detection of actionable lesions.

### Exploratory

The aim is to improve our understanding of disease progression and validate modalities for the early detection of pancreatic cancer. Disease progression in individuals who have pancreatic cancer will be studied based on different types of genetic predispositions and how they influence outcomes. The nature of progression and response to treatment in patients with diabetes mellitus will be compared in those with genetic predisposition for pancreatitis/cancer and those with spontaneous disease. Pancreatitis progression and complications will be compared between patients with different forms of predispositions. Different imaging techniques and forms of molecular analysis will be evaluated for the detection of pancreatic cancer in high-risk patient populations. We will create a bank of samples including DNA from affected and unaffected individuals.

## Statistical methodology

### Sample size

We aim to recruit 10 000 patients who will undergo secondary screening with follow-up until they are unsuitable (through comorbidity) to be considered for intervention should an actionable finding be discovered. It is expected that EUROPAC will find 1500 actionable findings. Most of these will be dysplastic pancreatic cysts which also require screening but also approximately 300 early-stage pancreatic cancers/high-risk precancerous lesions. Formal power calculations are difficult as the precise subgroups discovered and their interrelationships will be split in the patient population.

### Data analysis plan

Continuous variables will be presented as median with IQR unless clearly parametric, in which case mean and standard errors of means will be used. In order to carry out analysis of risk, nominal risk groups will be created; low, medium and high generally based on terciles of the population. Nominal data will be compared using Pearson χ^2^ or Fisher’s exact testing as appropriate.

Survival analysis will be performed by regression or the method of Kaplan and Meier. This will be carried out for age of onset of pancreatitis, diabetes, malabsorption, cancer, etc. These types of data will be tested for significance using log rank generally set at a two-sided 5% significance level.

Censorship will allow for competing risks, for example, with survival analyses of diabetes, malabsorption and cancer, we will censor at the time of surgery if individuals underwent a resection before event time. Pancreatic resection could preclude, and would certainly alter the subsequent risk of pancreatic cancer, which is why these two events will be considered as ‘competing risks’.

Time-varying covariates such as the age of diagnosis of diabetes will be used in the regression models rather than just binary factors (eg, diabetes yes, no).

Because the majority of patients die within a few months of diagnosis of cancer and age of diagnosis is subjective, death will be taken as the outcome measure.

The preferred regression method will be the Cox proportional hazards model, which will be extended to handle complications in the data such as frailties due to genetic similarities among family members, which are likely to lead to correlated survival times.

Our previous work has shown that for diseases such as pancreatitis and diabetes mellitus, the formula shown below can be used in regression models.

$$\lambda_{k}(t;X|Z_{k})=Z_{k}\lambda_{0}(t)e^{{\beta x}_{\text{ik}}(t)}$$

- λ_k_ (t) is the hazard function for the *k*^th^ proband.
- *i* represents each individual within the family.f
- x_ik_ denotes the value of the covariate (time-varying in the case of age of diagnosis of secondary diseases) of the *i*^th^ member from the *k*^th^ proband.
- Z_i_ are unobserved cluster-specific random effects (frailties), which may be due to shared environmental exposure or common genotype.

It is assumed that Z_i_ are independently and identically distributed random variables, which for convenience we will assume to follow a gamma distribution with mean 1 and variance θ. In multivariate analysis, correlation between pancreatic resection and the subsequent risk of pancreatic cancer will be taken into account using the model.

To test whether diseases such as diabetes are a symptom or predisposing factor for cancer, sensitivity analyses will be performed. The disease condition may be predisposing to cancer. In sensitivity analyses, it will be assumed the disease condition is a symptom if it was diagnosed within *x* years prior to diagnosis of cancer (*x*=1, 2, 3, 4, 5 or 10 in six separate analyses) to test whether this hypothesis is correct.

If cancer was diagnosed ≤*x* years after diagnosis of another disease, the date of cancer diagnosis will be brought forward to the date of diagnosis of the other disease, and disease status will be considered negative. If cancer is diagnosed >*x* years after the diagnosis of the other disease, the data will be unchanged. If a patient had <*x* years of follow-up from the date of diagnosis of the other disease but did not develop cancer, the dates of disease and cancer diagnosis will both be censored at the date of diagnosis of the other disease. If a patient had ≥x years of follow-up from the date of diagnosis of disease, data will be unchanged in the sensitivity analysis.

The link between genotype and phenotype will be assessed using association and linkage analysis. Hardy Weinberg equilibrium (p^2^+2 pq+q^2^=1) will be tested using the χ^2^ test of expected numbers of homozygote and heterozygote based on allele frequency.

Haplotypes will be divided into functional groups. Differences between groups of patients with these haplotypes will be tested using Mann-Whitney-U for continuous data χ^2^ or Fisher’s exact test for categorical data.

When testing the significance of specific genotypes, it will be assumed that non-carriers will be at no greater risk of outcome than the general population.

The R package ‘survival’, ‘cmprsk’ and ‘crrSC’ for Kaplan-Meier curves and Cox proportional hazards modelling will be used for cause-specific survival analysis.

Data will be available on reasonable request.

### Patient and public involvement statement

The EUROPAC Patient and Public Involvement (PPI) Group has contributed to the study conception and design, and they have continued involvement in the management of this study. There are lay representatives on the PPI Group to ensure that the study remains both acceptable and relevant to patients.

## Ethics and dissemination

### Ethical approval

EUROPAC is approved by the UK Health Research Authority with favourable opinion granted by the Yorkshire and Humber Research Ethics Committee on 30 September 2019. The details of this manuscript represent v.6 of the protocol approved on 30 September 2019. The study will be conducted in accordance with the Human Rights Act 1998, the Data Protection Act 2018, Freedom of Information Act 2000, the principles of GCP, the Declaration of Helsinki on biomedical research involving human volunteers (Hong Kong revision, 1989 and the 48th General Assembly, Somerset West, Republic of South Africa, October 1996, updated in October 2013) and the UK Policy Framework for Health and Social Care Research. Where individuals agree to take part in the study, they will be informed of how data are recorded, collected, stored and processed, and that data may be transferred to other countries, in accordance with UK General Data Protection Regulations.

### Data management considerations and data statement.

Data management will be through the University of Liverpool. The study has a dedicated trial manager, study coordinator, data manager and trial statistician and will be overseen by the Trial Steering Committee. Data management will be through the Progeny database and a dedicated Laboratory Information Management System (LIMS) within the GCP Laboratories at the University of Liverpool, linked by unique codes for each kit used at each time point from each patient, the code for which will be stored on Progeny and LIMS.

### Dissemination plan

Study results will be disseminated through presentations at national and international symposia and publication in peer-reviewed Open Access journals, where appropriate data will be made available via open-access repositories. We will work with charities, patient and public involvement groups and other relevant stakeholders to widely disseminate results and ensure that our findings are in an accessible format.

## supplementary material

10.1136/bmjopen-2025-100027 online supplemental file 1

10.1136/bmjopen-2025-100027 online supplemental file 2

10.1136/bmjopen-2025-100027 online supplemental file 3

10.1136/bmjopen-2025-100027 online supplemental file 4
