# Supplementary material for: European Registry of Hereditary Pancreatic Diseases (EUROPAC): protocol for primary and secondary screening in individuals with inherited pancreatic disease syndromes for pancreatic ductal adenocarcinoma and complications of other pancreatic diseases
Source: BMJ Open. 2025 Apr 3;15(4):e100027. doi: 10.1136/bmjopen-2025-100027 (PMC11969613; doi:10.1136/bmjopen-2025-100027)
Supplement: online supplemental file 1 [file bmjopen-15-4-s001.pdf]

EUROPAC
**The European Registry of Hereditary Pancreatic Diseases**
EUROPAC Office, 3rd Floor William Henry Duncan Building, 6 West Derby Street, University of Liverpool, L7 8TX
Tel: +44 (0)151 795 1256 email: europac@liv.ac.uk www.europactrial.com

# Participant Information Sheet

## The EUROPAC Study of Inherited Causes of Pancreatitis

**Version 7.0 dated 05/03/2024.**

## Background

Pancreatitis is a relatively common and potentially serious condition. Some of the causes of pancreatitis are known (e.g. excessive alcohol consumption and gallstones); however, in some individuals, this is less clear. Occasionally, there are multiple cases of pancreatitis in the same family and this suggests the disease is genetically determined. We do know that in some families individuals are very likely to develop pancreatitis and this type of pancreatitis is known as Hereditary Pancreatitis (HP). Hereditary conditions are caused by changes in genes, the genetic 'blueprints' that make the proteins of our body.

In other individuals where there are occasional cases of pancreatitis in the family, a genetic link is less clear and we do not describe this as HP; there may well still be a genetic element, but the cause of pancreatitis will be more strongly influenced by the environment (e.g smoking, alcohol, diet etc) of the individual. This type of pancreatitis is called familial pancreatitis. Finally, some individuals may develop pancreatitis with no obvious cause and this is known as idiopathic pancreatitis and may have a genetic component.

There are a variety of genes that may cause pancreatitis, both known and unknown. The known ones include the cationic trypsinogen gene (PRSS1) the SPINK 1 gene and the cystic fibrosis gene (CFTR). If you tested positive for the cystic fibrosis gene, this would NOT mean that you have cystic fibrosis or that you are at risk of developing it, but it MAY partly explain why you have pancreatitis.

## What Is the Study?

We are working together with a group of European pancreas specialists and would like to ask you to join a study centred in Liverpool, UK. We want to investigate why you or other members of your family could be at increased risk of pancreatitis and try to understand how the condition progresses over time.

## What is Involved Now?

We need about one hour of your time to complete some questionnaires and to then arrange to have a small blood sample taken. You can easily complete the questionnaire at home, whilst the blood sample will be taken after you have spoken to an expert on pancreas diseases and ideally a Geneticist (Doctor specialising in diseases associated with genes). This will usually take place at your local pancreas centre.

The questions will mainly be about how pancreatitis has affected you, any treatment or operations that you have had in the past, as well as lifestyle questions such as smoking and alcohol consumption. We will also ask about other members of your family and whether they have been diagnosed with pancreatitis.

The blood samples will be used to extract and store genetic material (DNA). These DNA samples may be stored for up to 25 years and will be tested to see if we can identify any genetic markers or altered genes for pancreatic disease, including genes that are associated with cystic fibrosis. If we identify a gene that is causing pancreatitis, we will suggest that unaffected family members seek genetic advice before proceeding with genetic tests, if these are appropriate.

Your participation in this study is entirely voluntary. You do not have to give a blood sample if you do not wish to, but you can still provide helpful information to the study. You can choose to withdraw at any time without explanation; this will not affect your treatment in any way. If you choose to withdraw, any information and samples of blood that have been given to us can be destroyed if you wish.

If appropriate, we would like as many of your family members as possible to become involved in the study, so that we can get as much information about pancreatitis as possible. It does not matter if they have pancreatitis or not, as all family information is important. If they are interested, your relatives should contact the EUROPAC office and we will go through the registration process with them.

## Medical Information

We would like those people joining the study to give permission for us to contact their doctors for more information about their medical history and if there has been any history of pancreatic problems. This will include asking for any surgical and pathological samples to be sent to Liverpool for further microscopic and genetic tests.

## Notification of Results

You will not be informed of the result of the blood tests taken for research purposes only. We will only provide results on genetic variations that are well-established and studied as pancreatitis-associated or pancreatitis-causing. This is because their clinical significance is better understood and they will be likely to have an impact on your future care and follow-up.

If you have given blood to be tested as part of routine management of your pancreatitis, you will be told the results by your local pancreatic specialist and/or a local clinical geneticist.

People are encouraged to join the study, whether they want to hear their results or not. The results of this research may help to guide us to know who is at increased risk from pancreatitis and to predict how their pancreatitis will progress or improve. We can then discuss whether further pancreatic screening tests are needed. If you decide not to hear the results of your tests, this will not affect your general medical care in any way.

## Confidentiality

This register will be held securely in Liverpool. No one outside of this study will have access to this data. Your GP can be informed that you have joined the study if you wish us to do so. With your permission, we would like to share information about any significant results with other members of your family for genetic counselling purposes only. We do aim to publish data that will include information from you, for example, we might publish your family tree but without names and dates of birth. It is EUROPAC policy to make scientifically irrelevant changes to these family trees to increase anonymity and certainly, no names will be published alongside.

A further risk is the publication of DNA sequences either in peer-reviewed journals or online. It is theoretically possible for an individual to trace back such data to you, but given this is contrary to research ethics, this is unlikely. We will choose our collaborators carefully on the basis of a reputation for good ethical behaviour, but it is impossible to know in advance which groups will be able to best use the information held by EUROPAC to further benefit patients. We will make every effort to reduce the risk of anyone being able to link your genetic data to you by avoiding simultaneously publicising DNA sequences along with other identifying features (age, ethnicity etc.).
You will be asked to explicitly consent to the publication of DNA sequences. If you choose not to consent to this we will avoid analysis of your DNA that could be used for such purposes. This will reduce the quality of the research that will be possible but we will respect your wishes.

## Adverse Effects

Apart from a small risk of bruising, the taking of a blood sample should not affect you. Genetic testing can reveal altered genes that can predict your susceptibility to certain illnesses in the future. We will only directly disclose test results on those people who have already had a pancreatic illness. This means we will not be reporting on predictive tests because hearing that you or members of your family are at increased risk of developing illnesses like pancreatitis can be worrying. If we find a faulty gene in you or your family, we recommend that any unaffected members of the family who are thinking of being tested should carefully weigh up the pros and cons of genetic testing in discussion with their GP in the first instance, who may recommend referral to an expert in Genetics, who will be able to offer further advice. If you, or a member of your family, are found to carry an altered gene, then there may be effects upon your ability to obtain health or life insurance, or employment in the future.

If taking part in this research project harms you, there are no special compensation arrangements. If you are harmed due to someone's negligence, then you may have grounds for a legal action but you may have to pay for it. Regardless of this, if you wish to complain, or have any concerns about any aspect of the way you have been approached or treated during the course of this study, the normal National Health Service complaints mechanisms should be available to you.

## Testing Children

Children who develop pancreatitis are more likely to carry faulty genes that cause their pancreatitis. In children who are showing symptoms of pancreatitis, a diagnostic test may be done at any age with the parent's consent. We would not consider testing any child (under the age of 16 years) unless they had symptoms suggestive of pancreatitis. General advice about lifestyle modification, such as the avoidance of alcohol and prompt attention to abdominal symptoms, can be given to all children at risk. If a child becomes symptomatic, then diagnostic testing can be offered at that point.

## Future Studies

As this research work proceeds, we hope to develop further research studies and recommendations. With your permission, we would like to contact you about these. Please keep in touch with the doctor or clinic that gave you this information sheet.

## PRECEDE Consortium

EUROPAC is part of the PRECEDE consortium and your coded information about you will be exchanged with collaborators in this consortium, which is coordinated from the United States by the group of Diane Simeone. The purpose of PRECEDE (Pancreatic cancer early detection) is to create a resource to drive research necessary for early detection and prevention of pancreatic ductal adenocarcinoma. We will therefore provide data and samples to support them. PRECEDE offers the option of a baseline visit and up to two visits per year. This is beyond the normal screening offered by EUROPAC and represents a different research emphasis. If you do not wish your data and samples to be shared across the PRECEDE consortium, you can indicate this on your consent from, but if you do wish for your data to be shared with PRECEDE, you may be offered the more frequent visits suggested by their protocol. This will not

indicate anything about your particular cancer risk and will merely be in order to provide consistent data across the international collaboration.

| | |
|---|---|
| Name of controller and contact details | William Greenhalf, University of Liverpool<br>Tel.0151 795 8030 |
| Purposes of the processing, as well as the legal basis | To assess disease progression of individuals and risk of disease in individuals or on a family basis. The legal basis is that an individual has given clear consent for us to process their personal data for a specific purpose |
| The legitimate interests of the controller or third party, where applicable | The controller is part of the research team and is an employee of the University of Liverpool, employed to carry out research for scientific and patient benefit |
| The categories of personal data concerned | 'sensitive', genetic, health, gender |
| The recipients or categories of recipients of the personal data, if any | Academics and clinicians |
| The period for which the personal data will be stored | We intend to store data for at least 25 years |
| The data subject's rights under GDPR | Under the GDPR, individuals can exercise:<br><br>• the right to be informed<br>• the right of access<br>• the right to rectification<br>• the right to erasure<br>• the right to restrict processing<br>• the right to data portability<br>• the right to object to processing<br>• the rights in relation to automated decision making and profiling |
| The right to lodge a complaint with the ICO | You have the right to lodge a complaint with Independent Commissioner's Office |
| The source from which the personal data originate, and if applicable, whether it came from publicly accessible sources | From your questionnaires and from family history questionnaires filled out by other members of your family. From your medical records and from cancer registry and other sources of family history data. |
| Whether the provision of personal data is part of a statutory or contractual requirement or obligation and possible consequences of failing to provide the personal data | This is not applicable to research |
| Any automated decision-making, and, meaningful information about the logic involved, as well as the significance and the envisaged consequences of such processing for the data subject | This study is for research purposes, but automated data analysis may be used and reported back to you or your clinicians (if appropriate) |
| How appropriate or suitable safeguards are achieved in relation to any personal data transferred out of Europe | All data transferred outside of Europe will be fully anonymised to the recipient |
