# Supplementary material for: European Registry of Hereditary Pancreatic Diseases (EUROPAC): protocol for primary and secondary screening in individuals with inherited pancreatic disease syndromes for pancreatic ductal adenocarcinoma and complications of other pancreatic diseases
Source: BMJ Open. 2025 Apr 3;15(4):e100027. doi: 10.1136/bmjopen-2025-100027 (PMC11969613; doi:10.1136/bmjopen-2025-100027)
Supplement: online supplemental file 2 [file bmjopen-15-4-s002.pdf]

The European Registry of Hereditary Pancreatic Diseases
EUROPAC Office, 3rd Floor William Henry Duncan Building, 6 West Derby Street, University of Liverpool, L7 8TX
Tel: +44 (0)151 795 1256 email: europac@liv.ac.uk www.europactrial.com

Participant Information Sheet

The EUROPAC Registry of Familial Pancreatic Cancer

Version 7.0 dated 05/03/2024

Background

Pancreatic cancer is the tenth most common cancer in the UK with around 10,000 people diagnosed each year. Pancreatic cancer has the lowest survival of all common cancers, with less than 1 in 4 surviving more than 1 year after diagnosis and less than 1 in 14 surviving beyond 5 years. In most cases, we do not know why pancreatic cancer develops, but we do know of risk factors that increase the likelihood of developing the condition. These factors include age, tobacco use, obesity and genetic factors. Having any one, or a combination of these does not mean you will definitely develop cancer.

Pancreatic cancer is a difficult cancer to diagnose, with less than 1 in 5 people being diagnosed at an early stage when treatment is most likely to be successful. Unfortunately, only 1 in every 10 people with pancreatic cancer will receive potentially curative surgery, only 2 in 10 will receive chemotherapy and 7 in 10 will not receive any active treatment. Nearly half of pancreatic cancer cases are diagnosed through an emergency presentation, where the one-year survival rate is only 12% which is three times lower than people diagnosed through a GP referral.

Most cases of pancreatic cancer appear out of the blue, with no previous family history of the condition. There are, however, families where there is more than one case of pancreatic cancer. This could be entirely coincidental but, in some cases, pancreatic cancer develops in these families because of a faulty gene that is inherited by affected individuals (familial pancreatic cancer).

Every organ in your body is made up of millions of cells. The growth of these cells is normally very carefully controlled; cancer develops when these cells grow out of control, forming a lump. Cell growth is controlled by the genes within each cell and damage to these genes can result in loss of control of how your cells divide. In many cancers, we know which genes have been damaged to cause that specific cancer, but in the majority of families with a history of pancreatic cancer, we do not yet know the causative gene.

Through years of dedicated research into pancreatic cancer, we now know some of the genes that cause pancreatic cancer to develop, however, we are still a long way from fully understanding the genetic cause of inherited pancreatic cancer.

We want to further our understanding of the genetic cause of pancreatic cancer. We want to understand the role of known genes in the development of pancreatic cancer and also want to search for other genetic causes of pancreatic cancer that have not yet been discovered.

As members of your family have been affected by pancreatic cancer, we would like to ask for your help in further studies to look at the causes of the disease. We hope to identify risk factors that will make us better able to make recommendations to you and others about the cause of pancreatic cancer. We want to be able to diagnose pancreatic cancer at a much earlier stage than we are able to at present and find out how we could improve screening for pancreatic cancer in people who are considered to be at a higher risk of developing it.

What Is the Study?

EUROPAC work with a group of pancreas specialists in the UK and Europe. All work is coordinated through the team based at the Royal Liverpool Hospital and the University of Liverpool. Since 1997, we have maintained Europe's largest database of families affected by pancreatic cancer. This has allowed us to further understand to what extent a family history of pancreatic cancer has on your individual lifetime risk of developing pancreatic cancer.

Every participant who registers with EUROPAC has their personal and family history added to our database. Once all the necessary information is available, we review your case in a steering meeting held weekly and determine your individual lifetime risk of developing pancreatic cancer and your eligibility to enter the second part of our study.

We also ask for a blood and urine sample from every person who registers with EUROPAC. We will take 2 samples of blood and a urine sample. One blood sample and the urine sample are used by our researchers and collaborators to further understand pancreatic cancer. The second blood sample is stored and used in the future to identify markers of early pancreatic cancer. Providing these samples is different to anything that a doctor would ask from you, as the results from these tests will not produce any results of clinical significance to yourself, so we do not generate any results that we can give back to you from this. Instead, the samples will allow for tests to be performed in scientific laboratories to further our understanding of pancreatic cancer.

If you are not considered to be at a higher lifetime risk of developing pancreatic cancer, we will write to you to let you know this and reassure you that there is no reason to be concerned by your family history. We will keep all the details of you and your family on our database and, should there be any new developments in your family, we ask that you contact us to let us know, at which point we will determine whether this has any significant impact on your lifetime risk of developing pancreatic cancer.

If you are considered to be at a higher lifetime risk of pancreatic cancer based on your family history, we will be in touch with further information about the next stage of our research project.

What is Involved Now?

Should you wish to register your family details with EUROPAC, we need you to complete a questionnaire that will give us all the information we require to enter your family on to our database. The questionnaire is all about you, your lifestyle and relevant medical history and asks about all of your relatives, whether they are alive or not, any relevant medical history and the cause of death of any deceased relatives.

We want the information we put in our database, and any advice that we give to you and your family, to be as accurate as it possibly can. We will not proceed with your registration unless we have all the necessary information that we need to do so. The most important part of this process is to confirm the information you are giving us about your affected relatives. We require confirmation of diagnosis for any relative who has been affected by pancreatic cancer or who has a relevant genetic syndrome. We can only accept confirmation in the form of:

- A letter from a pancreatic specialist, clinical geneticist or GP with all of your relative's details stating the diagnosis of pancreatic cancer or a relevant genetic syndrome.
- A death certificate stating pancreatic cancer as the cause of death

Sample donation

As mentioned above, we ask for a voluntary donation of blood and urine as part of your registration with EUROPAC. If you would prefer to give saliva or cheek swab samples we can arrange for this to happen. It is unlikely that the results of any tests done on these samples will be of any clinical significance to you or your family.

Occasionally, we approach our participants to ask for an additional blood sample to allow living cells to be stored indefinitely in the laboratory. This is separate to the blood and urine samples you may have already provided to assist us in our research. The immortalised cells can be stored frozen in a cell bank and used in future research projects. The samples collected will be under the custodianship of the University of Liverpool and participants will not have a personal claim on any commercial benefit that may arise from this study. We will destroy any samples received from individuals who later choose to withdraw from the study.

If during your time on the registry, you have surgery for pancreatic disease or another condition, we would like you to donate any tissue at that time for our research. You will be asked for this permission should that event arise.

The samples obtained from you will be used for future research without further consent. Stored samples may be released to researchers or agents for research or commercial purposes. Despite every effort to anonymise any genomic sequences, it is theoretically possible to identify you from your DNA sequence. You will have the right at a later date to request destruction of your samples and/or data from them. However, this may not always be possible if data has already been published or if samples from you may have been mixed with samples from other individuals.

Research Sample Results

Personal research results of genetic tests are not released to any study participants. However, we will notify a participant of any significant findings that may relate to their family, if they consent for us to do so on the study consent form. It is important to note that any tests performed on a research basis are not validated for use in clinical practice. If you hear that potentially significant findings have been made from this research, we suggest that you seek advice from a geneticist before proceeding with any independent tests for yourself.

In the unlikely event of identifying a clinically significant result and only if we have your permission to do so, we will contact you to inform you of this. If you do not consent to us disclosing any clinically significant results, you can still be part of the study and we will avoid any form of analysis that might inadvertently reveal something of known clinical significance to you or your family. Choosing not to know your test results from this research study will not have any impact on your general medical care and will not affect your health insurance status (as stated by the Association of British Insurers). However, we are unable to offer the same advice to anyone recruiting to EUROPAC from outside of the UK and suggest that you check your legal position prior to registering with us.

Confidentiality

The EUROPAC database is held securely on a locked computer at the University of Liverpool. No one outside of this study will have access to this data. No personal or identifying details will be disclosed to anyone outside of the EUROPAC team. With your permission, we would like to share information about any significant results pertaining to their lifetime risk of pancreatic cancer with other members of your family. All other personal information will be handled in a confidential manner. Results from the EUROPAC study will be published in scientific literature periodically. This may involve the inclusion of your family tree. Should we do so, all identifiable information will be removed and small, irrelevant changes may be made to preserve the anonymity of you and your family, this will have no impact on the scientific integrity of the published data.

DNA sequences will also be periodically published, either in peer-reviewed journals or online. It is theoretically possible for an individual to trace back such data to you, but it is very unlikely for this to happen as it is contrary to the ethical principles related to scientific research. Our collaborators have been chosen carefully based on their reputation for good ethical behaviour. Any data shared with our collaborators will be in coded form, which allows for your data to be transferred to our collaborators in the safest way possible. To further reduce the risk of any published genetic data being traced to a EUROPAC participant, DNA sequence will not be published alongside other identifying features.

You will be asked to explicitly consent to the publication of DNA sequences. If you choose not to consent to this we will avoid analysis of your DNA. This will reduce the quality of the research that will be possible but we will respect your wishes.

Adverse Effects

We recognise that this can be a worrying process to go through. Hearing that you or members of your family are at increased risk of developing illnesses like pancreatic cancer can be worrying. If you or any of your family members are struggling to process the information provided as a result of registering with us, please contact the EUROPAC office and we can arrange to talk through this with you. However, as we are not able to formally provide specific genetic counselling, we may also suggest talking to your GP or a clinical geneticist for further information.

Taking a blood sample from you should be a straightforward process and not affect you in any way. Occasionally, superficial bruising can occur around the area where the sample has been obtained. If an attempt to take blood from you is unsuccessful, this will not impact your registration with EUROPAC.

Genetic testing can reveal altered genes that can predict your susceptibility to certain illnesses in the future. We will only disclose the results of genetic tests that may be relevant to the family to participants who request us to do so.

Although every effort is made to preserve your confidentiality and protect your anonymity, it is theoretically possible for collaborators to identify you from information shared with them.

PRECEDE Consortium

EUROPAC is part of the PRECEDE consortium. Your coded information will be exchanged with collaborators in this consortium, which is coordinated from the United States by the group of Diane Simeone. The purpose of PRECEDE (Pancreatic cancer early detection) is to create a resource to drive research necessary for the early detection and prevention of pancreatic ductal adenocarcinoma (a specific and the most common type of pancreatic cancer). We will therefore provide data and samples to support them. If you do not wish your data and samples to be shared across the PRECEDE consortium you can indicate this on your consent form.

Future Studies

As this research work proceeds we hope to develop further research studies and recommendations. With your permission we would like to contact you about these; you can state whether you would like us to do this on your consent form.

Further information

If you require any further information or have any questions that have not been answered in this document, please contact the EUROPAC office and we will answer any queries you may have.

Further information about pancreatic cancer can be obtained from the Pancreatic Cancer UK website (www.pancreaticcancer.org.uk)

Data Protection

Name of controller and contact details	William Greenhalf, University of Liverpool<br>Tel.0151 795 8030
Purposes of the processing, as well as the legal basis	To assess disease progression of individuals and risk of disease in individuals or on a family basis. The legal basis is that an individual has given clear consent for us to process their personal data for a specific purpose
The legitimate interests of the controller or third party, where applicable	The controller is part of the research team and is an employee of the University of Liverpool, employed to carry out research for scientific and patient benefit
The categories of personal data concerned	'sensitive', genetic, health, gender
The recipients or categories of recipients of the personal data, if any	Academics and clinicians
The period for which the personal data will be stored	We intend to store data for at least 25 years
The data subject's rights under GDPR	Under the GDPR, individuals can exercise:<br>• the right to be informed<br>• the right of access<br>• the right to rectification<br>• the right to erasure<br>• the right to restrict processing<br>• the right to data portability<br>• the right to object to processing<br>• the rights in relation to automated decision making and profiling
The right to lodge a complaint with the ICO	You have the right to lodge a complaint with Independent Commissioner's Office
The source from which the personal data originate, and if applicable, whether it came from publicly accessible sources	From your questionnaires and from family history questionnaires filled out by other members of your family. From your medical records and from cancer registry and other sources of family history data.
Whether the provision of personal data is part of a statutory or contractual requirement or obligation and possible consequences of failing to provide the personal data	This is not applicable to research
Any automated decision-making, and, meaningful information about the logic involved, as well as the significance and the envisaged consequences of such processing for the data subject	This study is for research purposes, but automated data analysis may be used and reported back to you or your clinicians (if appropriate)
How appropriate or suitable safeguards are achieved in relation to any personal data transferred out of Europe	All data transferred outside of Europe will be fully anonymised to the recipient
