# Supplementary material for: European Registry of Hereditary Pancreatic Diseases (EUROPAC): protocol for primary and secondary screening in individuals with inherited pancreatic disease syndromes for pancreatic ductal adenocarcinoma and complications of other pancreatic diseases
Source: BMJ Open. 2025 Apr 3;15(4):e100027. doi: 10.1136/bmjopen-2025-100027 (PMC11969613; doi:10.1136/bmjopen-2025-100027)
Supplement: online supplemental file 3 [file bmjopen-15-4-s003.pdf]

EUROPAC
The European Registry of Hereditary Pancreatic Diseases
EUROPAC Office, 3rd Floor William Henry Duncan Building, 6 West Derby Street, University of Liverpool, L7 8TX
Tel: +44 (0)151 795 1256 email: europac@liv.ac.uk www.europactrial.com

Participant Information Sheet

Secondary Screening for Early Pancreatic Cancer in Hereditary Pancreatitis, Chronic Pancreatitis and Certain Cyst-like Growths of the Pancreas

Version 7.0 Dated 05/03/2024

What is the purpose of the study?

Following your registration with EUROPAC, we have been able to identify you as an individual who is at a higher lifetime risk of developing pancreatic cancer compared to the general population.

There needs to be a way of monitoring the pancreas of someone who has been considered to be at risk of pancreatic cancer, to allow for any cancers that may be developing to be diagnosed early on. Pancreatic cancer in its early stages does not usually cause symptoms and if it does, these are vague. Once pancreatic cancer starts to cause symptoms it is usually at an advanced stage and cannot be cured. However, if it is caught early, pancreatic cancer can be cured with a combination of surgery and chemotherapy. EUROPAC aims to develop a reliable way of screening high-risk individuals to increase the likelihood of diagnosing a developing pancreatic cancer at a stage where we can offer treatment.

Why have I been invited?

It is known that a diagnosis of hereditary pancreatitis, chronic pancreatitis or certain cyst-like growths of the pancreas increases the risk of pancreatic cancer.

You should have had a discussion about your personal circumstances that relate to your risk of pancreatic cancer compared to the general population and had the opportunity to discuss related issues and questions with a genetic counsellor and/or a consultant pancreatic specialist.

Do I have to take part?

You are under no obligation to participate in this part of the study. You have been offered the opportunity to take part based on our assessment of your risk. This information sheet will outline what will be involved should you agree to be involved. We will also offer you an appointment to discuss everything in our clinic or via telephone but please be aware that having this appointment does not commit you to taking part in the study either. If you choose not to become involved with the screening study, it does not affect your position with the registry in any way. If you wish to participate in the screening study, you can take up any or all of the proposed screening tools. If you become involved, you can change your mind at any time without giving a reason. This will not affect the standard of care you receive in any way.

UNIVERSITY OF LIVERPOOL
Supported by Pancreatic Cancer UK

What will happen if I take part?

Everyone who is offered the opportunity to take part in the screening will be seen in our EUROPAC clinic at the Royal Liverpool Hospital or offered a telephone appointment. At this appointment, we will discuss your personal and family history again and talk through the rationale and process of screening. You will have the opportunity to ask any questions and we will confirm your intention to take part and sign a consent form.

We work with a number of collaborators across the UK who can facilitate screening. If appropriate, we can refer you to a local screening centre who will offer you an appointment to meet them prior to screening.

After your appointment, we will start the screening process. This will involve blood tests and scans of your pancreas that will be performed: every six months, annually, or every other year depending on your risk. A set of baseline investigations will be arranged. Analysis of your pancreatic juice may be available. You can choose to take up any or all of the options. Once the results of your investigations are available, we will write to you with the outcome and inform you of any further action that may be required.

You will be seen in clinic or by telephone every year. At this appointment we will check for any changes in your medical history or if there have been any new developments in your family. We will talk through the results of your screening investigations and confirm with you your willingness to continue the screening process.

The blood tests and imaging can all be described as the best medical management for high-risk patients. The collection of pancreatic juice for molecular analysis is a research investigation. The findings are convincing within a laboratory setting but the molecular analysis has not been proven in living subjects in an ongoing trial.

There is no aspect of normal medical treatment that will be withheld as part of this trial and at the end of the trial it is expected that the results will be published in the scientific literature. You would not be identifiable to others as a result of any publications.

Expenses and Payments

There are no funds available for payments to those participating in this study.

What will I have to do?

Throughout the whole process, we ask that you keep the team updated with any changes in your personal or family history, specifically any new diagnoses of diabetes, pancreatitis or pancreatic cancer.

You will have to attend your screening hospital for your investigations and for follow-up clinic appointments. Your investigation appointments may come through at different times and we ask that you contact the relevant departments to arrange for them to take place on the same day should you want this to be the case. Your delegated Surveillance Navigator can also assist with arranging appointments.

What is being tested?

None of the blood tests or imaging techniques are ideal. The aim is to see what combination of tests gives the best chance of picking up pancreatic cancer early and the least chance of missing one.

The methods that can be taken up include:

Blood Tests
HbA1c
CA19-9

Imaging
Computed Tomography (CT)
Magnetic Resonance Imaging (MRI)

Molecular Methods
Oesophago-gastro-duodenoscopy (OGD) to allow molecular analysis of pancreatic juice

The HbA1c is a blood marker used in the diagnosis and management of diabetes. Your blood sugar levels are regulated by a hormone called insulin which is produced by your pancreas. Research has suggested that new-onset diabetes can be due to early pancreatic cancer. However, we do not know how reliably HbA1c can be used to detect early pancreatic cancer.

The CA19-9 is a tumour marker. It is used once a diagnosis of pancreatic cancer has been made to plan treatment and to monitor for recurrence or progression of the cancer. It is not always elevated in people with pancreatic cancer and can be elevated for reasons other than pancreatic cancer. It is not known whether CA19-9 can be used as a way of detecting pancreatic cancer in its early stages.

The CT scan uses X-rays to generate detailed images of your abdominal organs. It is commonly used to assess pancreatic cancers. It will require a dose of contrast to be given into your vein and you will be asked to fast before the scan.

The MRI is another type of scan used to visualise your pancreas and surrounding structures. It involves lying on a bed which moves you in and out of a tunnel. It takes approximately 30 minutes to complete the scan. We will not use MRI if you do not have additional risk factors for developing pancreatic cancer, we will talk about this in clinic before starting your screening.

The OGD is a procedure that involves passing a camera through your mouth and down your food pipe (oesophagus) to get into your stomach and duodenum (the first part of your small bowel/intestine). It is used to collect the juice that drains into your duodenum from your pancreas. Changes in your pancreatic juice DNA (the genetic material that has come from the cells in your pancreas) could signify a possible early tumour that cannot be seen on the imaging investigations. These DNA changes (if found) will have arisen during your lifetime and will not be the sort of DNA changes that can be passed on to your children.

During the OGD we may use an artificial version of a human gut hormone called ‘secretin’. Secretin is produced naturally within the gut to stimulate the release of pancreatic juice and aid the digestive process. We use a man-made

version of this hormone, which is given into a vein, to stimulate the release of pancreatic juice which is then collected within the small intestine. The juice collected in this way will undergo DNA analysis. If we plan to use secretin during your procedure, then you will be informed beforehand.

If the investigations suggested a possible problem, then your case would be discussed at a special meeting of pancreatic experts to decide on a plan of treatment. They may recommend continued surveillance, further tests to clarify the findings or even an operation if a growth were present that could be removed.

What are the alternatives?
You can take up any or all of the screening investigations. You could choose not to participate in the screening study and continue as you have done until today.

What are the possible disadvantages of taking part?

All the screening methods have advantages and disadvantages.

The blood tests are very low risk and a serious complication would be very rare. All the equipment used will be sterile, single-use equipment in common use within the NHS. A bruise would be a relatively common complication.

The CT scan builds up an image by passing radiation through the body. It is known that moderate and high doses of radiation are damaging and at worst could even cause a cancer. The risk of cancer from each CT scan is described as low and calculated at somewhere between a one in a thousand and one in ten thousand chance. A single CT scan of the abdomen has been calculated to be the equivalent of a few years of normal background radiation. For this reason, we only conduct one baseline CT scan.

The MRI scan uses strong magnets to generate the images. If you have any metallic implants in your body (such as coils in the blood vessels around your brain or a pacemaker) it is very unlikely that you will be able to have an MRI. We will talk about this in clinic. You will lie on a bed and pass through a tunnel during the scan, if you have claustrophobia or become anxious in enclosed spaces you may not tolerate the MRI.

The OGD involves passing a camera through your mouth, down your food pipe (oesophagus) and into the stomach. The throat is numbed with local anaesthetic, and you are sedated during the procedure, so it is normally well tolerated. There is a risk of causing damage to the oesophagus or stomach during the procedure. This is a rare complication (and can very rarely be fatal) that occurs at a rate of between one in a thousand and one in ten thousand procedures. If this were to occur it can be treated and may (but doesn't always) require an operation to repair it.

Other disadvantages

You may find that having screening investigations makes you more anxious about your health and your cancer risk. This is difficult to predict. Other people find having the investigations reassuring as it helps to put their minds at rest.

Any of the blood and imaging methods could detect a problem that is unrelated to pancreatic cancer. CT scans are very effective at detecting problems within the body and there is always the possibility of finding something unexpected. If this falls within the expertise of the clinician who would oversee the screening process for you, they would deal with this. If appropriate, you would be referred to another specialist. Conversely, all the investigations

performed are targeted towards your pancreas so there is a small chance that conditions developing outside of your pancreas may not be detected as this is beyond the scope of the imaging being performed.

It is possible that the screening investigations could detect a problem with the pancreas. Growths can be "benign", (for example, inflammation or scarring) or could be a cancer. It is difficult to tell the difference between the two without performing further investigations. This may include further scans or biopsies of an area of concern. If a growth is found and it is possible to remove it, your consultant would discuss your options with you in the clinic and an operation may be offered.

Harm to the unborn child

Men

Damage to male sperm as a result of this study should not occur as any radiation exposure would affect the upper part of the abdomen, well away from the genital organs. However, it is recommended that you avoid starting a pregnancy with your partner in the three months after having investigations that involve ionising radiation.

Acceptable methods of contraception for men include:

- Use of a condom
- In addition to a condom, male participants without a vasectomy must assure that their female partner uses another form of contraception such as an intra-uterine device (IUD), spermicidal foam/gel/film/cream/pessary, diaphragm with spermicide, oral contraceptive, injectable progesterone, sub-dermal implant, or tubal ligation if your female partner could become pregnant.

Your partner is not required to use another form of contraception if they are of non-childbearing age.

Please inform the EUROPAC office if your partner becomes pregnant during the course of the study.

Women

It is possible that the radiation exposure involved with this study could damage an unborn child.

Being pregnant or actively trying to become pregnant will not affect your entry into the study, but it is important that you defer any investigations, particularly those involving ionising radiation, until after the pregnancy and you are no longer breast feeding.

If there is any doubt about possible pregnancy you may be asked to have a pregnancy test.

Women who could become pregnant should use an effective form of contraception during the course of this study.

The acceptable methods include:

- Tubal ligation
- Physician documented placement of an intra-uterine device (IUD)
- Diaphragm with spermicidal foam/gel/film/cream/pessary
- Condom with spermicidal foam/gel/film/cream/pessary
- Male partner who has had a vasectomy
- Hormonal contraceptives

You are not required to use any form of contraception if you are of non-childbearing age.

Any woman who becomes pregnant during the course of the study should immediately inform the EUROPAC office.

What are the possible benefits?

Hereditary pancreatitis, chronic pancreatitis and certain cyst-like growths of the pancreas carry an increased risk of developing pancreatic cancer. If you develop pancreatic cancer during the period of screening, it is hoped that it would be detected and earlier surgical treatment may achieve a cure.

What happens when the research stops?

This depends on the outcome of the study. After the end of the term of the study, if an effective screening protocol has been developed, it is likely to continue, funded by the NHS. If the proposed investigations are shown to be ineffective at accurately diagnosing early pancreatic cancer at a time when it can be cured, screening would be unlikely to continue to be offered in its present form through the NHS. Individual consultants may continue to offer some ongoing surveillance via the NHS within families with an elevated risk, depending on their own personal practice.

What will happen if I don't want to carry on with the study?

You can withdraw from the study at any time. We will confirm your intention to remain within the study before booking your next set of investigations when we see you in clinic. Should you withdraw and later want to re-join the screening program, please inform the EUROPAC office or local screening centre and this can be restarted assuming that the trail is still actively recruiting. Withdrawal from the study would not affect your clinical care. Information we have collected before your withdrawal will not be destroyed or erased and will not be considered when the screening process is analysed.

If you wish to withdraw any stored blood or tissue samples, we will do this wherever possible. Sometimes samples will have been combined with others. If all the material that you originally donated cannot be destroyed, all reference to you will be removed from any database relating to the sample. This means that the sample will be anonymised and will not be traceable back to you under any circumstances.

If your heath deteriorates during your time on the study and we feel that the risks of screening outweigh any benefit from you continuing we will recommend that you withdraw from the screening program but remain on our registry.

What if there is a problem?

Complaints

If you have a concern about any aspect of this study, you should ask to speak to a member the EUROPAC team who can advise on their complaints procedure. If you would rather not deal with the staff in the office, the clinical consultant overseeing your care or your General Practitioner (GP) should be contacted. If you would like to raise any queries or a complaint relating to research studies involving the University of Liverpool, please contact ethics@liverpool.ac.uk to discuss the issues further. If you do not feel that any of these routes is open to you,

you could complain to the chief executive of the trust via the patient advice liaison service (PALS) where you are being screened and the normal NHS complaints procedure would be followed.

Harm

If you were to come to any harm as a result of this study, NHS indemnity and insurance procedures apply. In the event that something does go wrong and you are harmed during the research and this is due to negligence, you may have grounds for a legal action for compensation against the University of Liverpool and the Liverpool University Hospitals Foundation Trust, but you may have to pay your legal costs. The normal NHS complaints mechanisms will still be available to you. NHS indemnity does not offer compensation for harm that is not the result of negligence.

NHS based research

NHS bodies are liable for clinical negligence and other negligent harm to individuals covered by their duty of care. NHS institutions employing researchers are liable for negligent harm caused by the design of studies they initiate.

Will taking part be confidential?

All data handling and storage will be in accordance with Caldicott principles and/or the Data Protection Act 2018 (GDPR). All information collected about you during the course of the research will be kept strictly confidential and any information about you that leaves the hospital will have your name and address removed so that you cannot be recognised. If you participate, you have the right to check the accuracy of data held about you is correct. Data is collected from hospital computer systems and case notes and is stored on the main EUROPAC registry database, which is password-protected. Only the EUROPAC team have the passwords to access the data. The data will be used to allow statistical analysis for any detectable changes in patients that both develop and do not develop pancreatic cancer. After the period of this study, data may be retained, subject to further ethical committee approval. Any destruction of data will be performed securely.

Involvement of the General Practitioner

Results would normally be communicated to your GP unless you explicitly state at the appropriate point on the consent form that they should not be. As a general principle, it is important that your GP has as much information that relates to your healthcare as possible. This enables them to make accurate decisions relating to your health.

What will happen to any samples that I give?

We will ask you for blood samples to use for analysis directly carried out by your screening centre. The blood sample used directly for your screening will be processed and the blood will be stored for less than a month afterwards before being destroyed in accordance with NHS procedures.

Duodenal aspirate gathered at OGD will be stored in the Department of Molecular and Clinical Cancer Medicine at Liverpool University for ten years. If changes were detected in your pancreatic juice you may be asked for a further blood sample for research, which will be stored for a maximum of 10 years. This blood sample would be used to see if the changes detected in your pancreatic juice could be found in your blood. It is very unlikely that this will have any immediate consequence for you, but it may allow us to develop improved screening systems that could be applied later. If you were ever to require an operation, we would ask for your permission to retain small amounts of tissue.

Any tissue or blood storage complies with both the Data Protection Act (DPA) 2018 and the Human Tissue Act (HTA) 2004. Information is coded and kept isolated from the internet on password-protected databases. Destruction of any data will be performed in a secure fashion.

Will any genetic tests be done?

There will be no genetic (inheritable) testing done as part of this study. If genetic tests are available to you, this will have been discussed as part of your existing membership of the EUROPAC registry. Your eligibility for genetic testing in the future will not be affected by your inclusion in the screening study and any future discussions between you and members of the EUROPAC study group regarding genetic testing will be completely independent of the screening study.

The molecular analysis of the DNA in your pancreatic juice is to look for cancer related changes. Detection of these changes will have no implications for the health of your children or other relatives. As these changes will not have been inherited and cannot be passed on to your children, tests for these changes would not normally be described as genetic tests.

What will happen to the results of this study?

You will be made aware of the results of your screening blood and imaging investigations during the course of the study. Any results from the molecular analysis of your pancreatic juice would only be given to you if you expressly request them. Although these tests may be used by your specialist to guide the timing of the other investigations, the significance of these test results is not clear at present and they should be considered as research data.

The overall results of the research project will be made available at the end of the study (in an anonymised form) via publications in scientific journals and presentations at scientific meetings. Once results have been published, copies will be available from the EUROPAC office and anonymous non-sensitive information may be made available on the EUROPAC website.

Who is organising and funding the research?

The sponsors of this study are The Royal Liverpool University Hospital and The University of Liverpool. The study is being funded by Pancreatic Cancer UK (PCUK), Cheshire and Merseyside Cancer Alliance and NHS England. The research team do not have any financial interest in the study and there are no financial payments or inducements of any kind for recruiting participants to the study.

Who has reviewed the study?

All research in the NHS is looked at by an independent group of people called a Research Ethics Committee to protect your safety, rights, wellbeing and dignity. This study has been reviewed and given favourable opinion by the Yorkshire and Humber Research Ethics Committee.

PRECEDE Consortium

EUROPAC is part of the PRECEDE consortium and your coded information about you will be exchanged with collaborators in this consortium, which is coordinated from the United States by the group of Diane Simeone. The purpose of PRECEDE (Pancreatic cancer early detection) is to create a resource to drive research necessary for early detection and prevention of pancreatic ductal adenocarcinoma. We will therefore provide data and samples to support them. PRECEDE offers the option of a baseline visit and up to two visits per year. This is beyond the normal screening offered by EUROPAC and represents a different research emphasis. If you do not wish your data and samples to be shared across the PRECEDE consortium you can indicate this on your consent from, but if you do wish for your data to be shared with PRECEDE you may be offered the more frequent visits suggested by their protocol. This will not indicate anything about your particular cancer risk and will merely be in order to provide consistent data across the international collaboration.

GDPR, Rights and Responsibilities

Name of controller and contact details	William Greenhalf, University of Liverpool Tel.0151 795 8030
Purposes of the processing, as well as the legal basis	To assess disease progression of individuals and risk of disease in individuals or on a family basis. The legal basis is that an individual has given clear consent for us to process their personal data for a specific purpose
The legitimate interests of the controller or third party, where applicable	The controller is part of the research team and is an employee of the University of Liverpool, employed to carry out research for scientific and patient benefit
The categories of personal data concerned	'sensitive', genetic, health, gender
The recipients or categories of recipients of the personal data, if any	Academics and clinicians
The period for which the personal data will be stored	We intend to store data for at least 25 years
The data subject's rights under GDPR	Under the GDPR, individuals can exercise:<br>• the right to be informed<br>• the right of access<br>• the right to rectification<br>• the right to erasure<br>• the right to restrict processing<br>• the right to data portability<br>• the right to object to processing<br>• the rights in relation to automated decision making and profiling
The right to lodge a complaint with the ICO	You have the right to lodge a complaint with Independent Commissioner's Office
The source from which the personal data originate, and if applicable, whether it came from publicly accessible sources	From your questionnaires and from family history questionnaires filled out by other members of your family. From your medical records and from cancer registry and other sources of family history data.
Whether the provision of personal data is part of a statutory or contractual requirement or obligation and possible consequences of failing to provide the personal data	This is not applicable to research
Any automated decision-making, and, meaningful information about the logic involved, as well as the significance and the envisaged consequences of such processing for the data subject	This study is for research purposes, but automated data analysis may be used and reported back to you or your clinicians (if appropriate)
How appropriate or suitable safeguards are achieved in relation to any personal data transferred out of Europe	All data transferred outside of Europe will be fully anonymised to the recipient
